# Synthesis of a Rivastigmine and Insulin Combinational Mucoadhesive Nanoparticle for Intranasal Delivery

**DOI:** 10.3390/polym16040510

**Published:** 2024-02-13

**Authors:** Tahereh Jamshidnejad-Tosaramandani, Soheila Kashanian, Isaac Karimi, Helgi B. Schiöth

**Affiliations:** 1 Nanobiotechnology Department, Faculty of Innovative Science and Technology, Razi University, Kermanshah 6714414971, Iran; t.jamshidnejad89@yahoo.com; 2 Laboratory for Computational Physiology, Department of Biology, Faculty of Science, Razi University, Kermanshah 6714414971, Iran; ikarimi2010@gmail.com; 3 Department of Surgical Sciences, Division of Functional Pharmacology and Neuroscience, Uppsala University, 62167 Uppsala, Sweden; 4 Faculty of Chemistry, Sensor and Biosensor Research Center (SBRC), Razi University, Kermanshah 6714414971, Iran

**Keywords:** Alzheimer’s disease, intranasal drug delivery, rivastigmine, insulin, co-delivery, *N*,*N*,*N*-trimethyl chitosan, mucoadhesive nanoparticles, combinational therapy

## Abstract

Efficient drug delivery remains a critical challenge for treating neurodegenerative diseases, such as Alzheimer’s disease (AD). Using innovative nanomaterials, delivering current medications like acetylcholinesterase inhibitors to the brain through the intranasal route is a promising strategy for managing AD. Here, we developed a unique combinational drug delivery system based on *N*,*N*,*N*-trimethyl chitosan nanoparticles (NPs). These NPs encapsulate rivastigmine, the most potent acetylcholinesterase inhibitor, along with insulin, a complementary therapeutic agent. The spherical NPs exhibited a zeta potential of 17.6 mV, a size of 187.00 nm, and a polydispersity index (PDI) of 0.29. Our findings demonstrate significantly improved drug transport efficiency through sheep nasal mucosa using the NPs compared to drug solutions. The NPs exhibited transport efficiencies of 73.3% for rivastigmine and 96.9% for insulin, surpassing the efficiencies of the drug solutions, which showed transport efficiencies of 52% for rivastigmine and 21% for insulin ex vivo. These results highlight the potential of a new drug delivery system as a promising approach for enhancing nasal transport efficiency. These combinational mucoadhesive NPs offer a novel strategy for the simultaneous cerebral delivery of rivastigmine and insulin, which could prove helpful in developing effective treatments of AD and other neurodegenerative conditions.

## 1. Introduction

Alzheimer’s disease (AD) is the primary cause of dementia among the elderly population, ranking as the fifth leading cause of death worldwide [1]. With the global aging population, the number of AD cases is projected to rise significantly in the future [2]. Currently, managing this complex, progressive neurodegenerative condition relies mainly on symptomatic therapy, aiming to restore neurotransmitter balance in the brain, but still with limited success [3]. Despite extensive research efforts and experience, existing therapeutic approaches, such as acetylcholinesterase inhibitors and *N*-methyl-d-aspartate receptor antagonists, following the conventional one-compound-one-target paradigm, have largely proven ineffective [4,5,6]. To halt AD progression, therapeutic agents may need to target the different underlying causes of the disease, including the accumulation of extracellular amyloid β (Aβ) plaques, intracellular tau neurofibrillary tangles, oxidative stress, inflammatory processes, metabolic abnormalities, impaired growth factors, synaptic dysfunction, neural loss, and particularly the inhibition of acetylcholinesterase [7,8]. Therefore, embracing a polypharmacology approach could be crucial for implementing more effective therapeutic strategies [9].

Acetylcholinesterase inhibitors can play a pivotal role in enhancing cerebral cholinergic neurotransmission by preventing the degradation of acetylcholine (ACh) in the synaptic cleft and inhibiting Aβ deposition [10,11,12,13]. Rivastigmine (Exelon^®^, Chicago, IL, USA), among the acetylcholinesterase inhibitors, has demonstrated the most significant therapeutic benefits, particularly when administered early and continuously [14]. This non-competitive, pseudo-irreversible inhibitor effectively targets both acetylcholinesterase and butyrylcholinesterase with a more selective action on cerebral acetylcholinesterase [10]. However, the use of rivastigmine faces several challenges, including poor penetration through the blood-brain barrier, limited bioavailability due to its hydrophilic nature, and extensive first-pass metabolism, resulting in a short plasma elimination half-life [14]. Moreover, oral administration of rivastigmine is associated with gastrointestinal side effects, such as nausea, diarrhea, vomiting, and hepatotoxicity [15]. It is also important to note that acetylcholinesterase inhibitors do not address other pathological mechanisms underlying the disease [9]. To address these limitations, the development of multifunctional drug delivery systems could become important. These drug delivery systems can incorporate non-overlapping and/or synergistic medications, enabling the targeting of additional pathomechanisms involved in AD. By employing combinational therapy modalities, these innovative drug delivery systems have the potential to improve disease management approaches significantly [16,17,18].

In addition to common acetylcholinesterase inhibitors, drug repurposing offers a safe and cost-effective prospect to improve symptoms of Alzheimer’s disease (AD) [19,20,21]. Intranasal insulin has emerged as a promising remedy for both the prevention and treatment of AD, owing to its role in normal memory functioning [22,23]. Recent studies have indicated a relationship between insulin resistance and the onset of neurodegeneration and have demonstrated that acute and prolonged intranasal insulin administration improves memory performance in healthy individuals and those affected by AD [24,25,26]. The administration of insulin through the nasal route enhances glucose uptake in the brain, promotes synaptic neuroplasticity, modulates the disposition of Aβ, and alleviates AD neuropathology [27]. Therefore, intranasal insulin administration can serve as a therapeutic approach alongside conventional AD therapies [28]. However, delivering large hydrophilic proteins, such as insulin, through the nasal mucosa presents significant challenges due to low drug retention time in the nasal cavity, mucociliary clearance, and potential enzymatic degradation [29]. Several previous reports have highlighted limitations associated with the intranasal delivery of insulin as a drug solution [30,31,32].

Innovative treatments using combination NPs have emerged as a promising approach for improving the management of AD through intranasal delivery, which offers higher drug accumulation in the brain compared to equivalent drug solution formulations and systemic approaches [33,34,35,36,37,38,39,40,41,42]. Among these approaches, derivatives of the chitosan (Cs) polymer, such as *N*,*N*,*N*-trimethyl chitosan (TMC), have garnered attention due to their positive biological features, including high mucosal absorption capacity, low toxicity, and ease of NP formation through ionic gelation [43,44]. In comparison to Cs, TMC exhibits excellent mucoadhesive properties and solubility in aqueous solutions across a wide pH range [15]. The administration of TMC-based intranasal drug delivery systems provides a non-invasive and powerful strategy for delivering drugs to the central nervous system (CNS), bypassing peripheral side effects [45,46,47]. Therefore, the aim of this study was to develop and characterize an innovative combination NP formulation incorporating rivastigmine and insulin within TMC-NPs, enabling the delivery of their daily therapeutic doses of 1.5 to 6 mg rivastigmine and 20–40 IU insulin. This study represents the first attempt to synthesize mucoadhesive TMC-NPs containing both rivastigmine and insulin using the ion gelation method, which offers a suitable combination therapy for intranasal administration.

## 2. Materials and Methods

### 2.1. Chemicals

Low molecular weight chitosan (Mw < 100 kDa), sodium tripolyphosphates (TPP), dialysis bag (mol. wt. cut-off: 14 KDa), potassium dihydrogen phosphate (KH_2_PO_4_), sodium hydroxide (NaOH), hydrochloric acid (HCl), sodium chloride (NaCl), methyl iodide, 1-methyl-2-pyrrolidinone (NMP), sodium iodide (NaI), iodomethane (CH_3_I), dimethyl ether, potassium chloride (KCl), sodium bicarbonate (NaHCO_3_), calcium chloride (CaCl_2_), potassium bromide (KBr) were purchased from Sigma-Aldrich (St. Louis, MO, USA). Double deionized water (DDW), methanol, and acetonitrile, all HPLC grade, were purchased from Sigma-Aldrich (St. Louis, MO, USA). A rivastigmine sample was received from Daroupakhsh Co. (Tehran, Iran), and human insulin was kindly provided by Ronak Daroo Co. (Tehran, Iran).

### 2.2. Methods

#### 2.2.1. Synthesis of TMC Chloride Polymer

The reductive methylation reaction in two steps was conducted on low molecular weight Cs to synthesize TMC based on the previously reported protocol [1], with some minor modifications. Briefly, 0.5 g of Cs were dispersed in 25 mL of 1-methyl-2- pyrrolidinone (NMP) at a constant temperature (60 °C) in darkness and stirred continuously until the complete dispersion of Cs (~12 h). Next, 40 mL aqueous NaOH solution (15% *w*/*v*) and 1.5 g of sodium iodide (NaI) were added to the container, followed by the addition of 40 mL of iodomethane (CH_3_I) in three steps. The solution was kept under stirring for more than 24 h. The product (*N*,*N*,*N*-trimethyl chitosan iodide, TMI) was precipitated using acetone, solved into 20 mL sodium chloride solution (NaCl, 10% *w*/*v*) to exchange the iodide (I^−^) with chloride (Cl^−^) ions, then poured into a dialysis bag (14 kDa) and immersed in DDW for three days to completely exchange the ions. The solution inside the dialysis bag was finally freeze-dried. The final TMC chloride, a water-soluble white powder, was used for Fourier-transform infrared (FTIR) spectroscopy (Bruker, Leipzig, Germany) and ^1^H Nuclear magnetic resonance (^1^H NMR) spectroscopy (Varian, Palo Alto, CA, USA) for polymer characterization and further NPs synthesis.

#### 2.2.2. Preparation of TMC-NPs

The TMC-NPs containing both rivastigmine and insulin were synthesized according to the ionic gelation method with a minor modification [2]. First, a premixed rivastigmine (6 mg/mL) and TMC aqueous solution (2 mg/mL), pH 5.5 ± 0.1, were prepared and stirred at room temperature. Second, a premixed insulin and TPP aqueous solution (both 1 mg/mL), pH 8.0 ± 0.1, were prepared. Then, TMC-NPs were obtained upon the addition of the premixed insulin and TPP solution to the premixed rivastigmine and TMC solution in a dropwise manner. The ratios of TMC/TPP and TMC/drugs were established according to the sets of optimization experiments, with the one-factor-at-a-time method, for variables of polymer, cross-linker, and drug ratios based on reported ranges in the previous studies [3,4,5,6,7,8,9]. The TMC-NPs were collected from the aqueous medium containing non-associated drugs using centrifugation (Sigma, VA, USA) at 20,000 rpm for 20 min at 4 °C and the supernatant was submitted to determine encapsulation efficiency (EE) according to equation (Equation (1)). The amount of both free drugs in the supernatant was measured using high-performance liquid chromatography (HPLC, Agilent, Waldbronn, Germany) simultaneously. The EEs of the rivastigmine- and insulin-loaded TMC-NPs were calculated as per the equation given below, with the measurements performed in triplicate and averaged.

EE = (Total drug − free drug)/(Total drug) × 100(1)

#### 2.2.3. Characterization of TMC-NPs

The characteristics of the TMC-NPs were studied first via dynamic light scattering (DLS, Malvern, Worcestershire, UK). Then, scanning electron microscope (SEM, TESCAN, Prague, Czech Republic, MIRA III model) at an operating voltage of 15 kV was used to examine the surface morphology of NPs and confirm the DLS data. Next, in vitro cumulative release profiles in simulated nasal fluid (SNF) (pH 6.5 ± 0.1), from 0 to 240 min, at 33 °C (nasal cavity temperature), diagrammed based on dialysis bag method for rivastigmine [3]. The samples were analyzed via HPLC (Agilent, Waldbronn, Germany) in the aforementioned conditions to determine the concentration of rivastigmine. Withdrawn samples were replaced with the same amount of fresh SNF each time to keep the experiment volume constant.

#### 2.2.4. Ex Vivo Diffusion of Drug Solutions and TMC-NPs

The ex vivo biologic membranes are valuable tools for predicting the capacity of NPs in drug diffusion [10]. Here, freshly excised sheep nasal mucosa was dipped immediately in Krebs–Henseleit buffer solution (pH 7.4). Cartilages were removed properly, and the mucosal membrane was cut off and washed with SNF (pH 6.5). An ex vivo drug diffusion study was bio-mimicked using a Franz’s diffusion cell. The excised sheep nasal mucosa was fixed on Franz’s cell. Ex vivo diffusion of the free drug was conducted by placing 3 mL of each drug solution (6 mg/mL for rivastigmine and 1 mg/mL for insulin) in the donor compartment of the cell onto the stabilized membrane. The receptor compartment was prefilled with SNF (pH 6.5, 33 ± 1 °C) and stirred during the experiment. In the case of NPs, the solution of TMC-NPs, equivalent to 6 mg rivastigmine and ~1 mg of insulin, was spilled on the membrane into the donor part of the cell. The samples from the receptor part were withdrawn at predetermined time intervals and substituted with fresh SNF. Withdrawn samples were filtered through a 0.2-µm syringe filter and analyzed using an HPLC (Agilent, Waldbronn, Germany) at 215 nm for the determination of both rivastigmine and insulin concentrations simultaneously. The percentage of drug diffusion was calculated from the calibration curve of both drug solutions in the SNF (pH 6.5).

#### 2.2.5. HPLC Analysis

**Figure 1:**
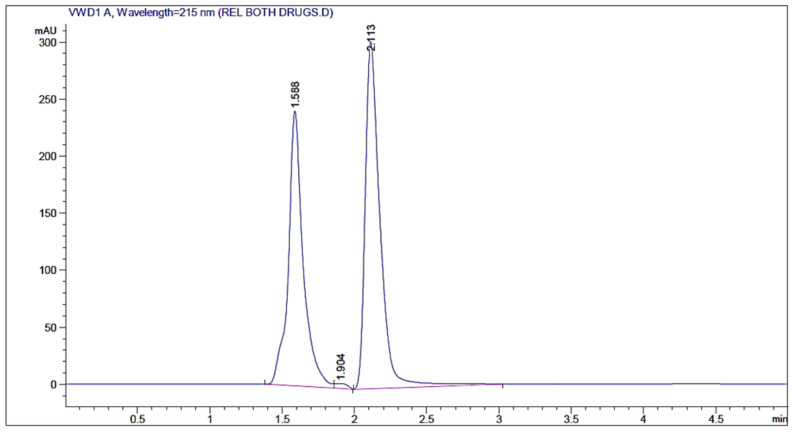
The chromatograms of both insulin (retention time = 1.588 min) and rivastigmine (retention time = 2.113 min) via the mobile phase of sodium sulfate buffer solution and acetonitrile mixture at a flow rate of 0.75 mL min^−1^, and the injection volume of 2 µL, monitored at UV wavelength of 215 nm. The peaks are represented in blue and the baselines are represented in pink.

All measurements of the rivastigmine and insulin were completed using HPLC (Agilent, Waldbronn, Germany). A filtered and degassed mixture of sodium phosphate buffer solution and acetonitrile was used as the mobile phase. The equipment consisted of a UV–Vis detector and reversed-phase column C18, 4 µm. The mobile phase was delivered at a flow rate of 0.75 mL min^−1^, the injection volume was 2 µL, the effluent was monitored at UV detection at 215 nm, and the retention times for insulin and rivastigmine were 1.588 and 2.113 min, respectively (Figure 1).

#### 2.2.6. The Evaluation of TMC-NPs’ Biocompatibility

Finally, the toxicity of TMC-NPs was considered on the sheep excised nasal tissue to verify the biocompatibility of NPs [11]. The obtained nasal tissue was socked in Krebs–Henseleit buffer solution (pH 7.4) at the local butchery. We considered a negative control (NC), PBS (pH 6.8 ± 0.1), a positive control (PC), isopropyl alcohol, and test samples, drug solutions mixture, and TMC-NPs solution (pH 6.00 ± 0.1) for the evaluation of the biocompatibility of TMC-NPs. The sheep nasal tissue was sectioned into four pieces, and each piece was soaked in the established amount of negative and positive controls and test samples for ~1 h on a laboratory shaker. Then, the samples were washed properly with distilled water and were fixed in 10% v/v formalin solution for a day, and embedded in paraffin. Then, the prepared samples were stained with hematoxylin-eosin (HE) and observed under an optical microscope (Olympus, Tokyo, Japan). To determine nasal toxicity, epithelial detachment (ED), mural destructive lesions of venules and arteries, as well as intercellular space widening (ISW) were assayed as the toxicity indicators.

#### 2.2.7. Statistical Analysis

We used SPSS version 20 (SPSS Inc., Chicago, IL, USA) to analyze the biocompatibility of the NP data and show the result as means ± SEM (standard error of the mean). The one-way analysis of variance (ANOVA) was employed to show the biocompatibility of the NPs in comparison to the solutions of both drugs: NC and PC groups. The post hoc Tukey’s HSD has been pursued when ANOVA indicated a significant difference (*p* < 0.05). In all statistical evaluations, a value of *p* < 0.05 was considered as significant level.

## 3. Results

### 3.1. Synthesis of TMC Polymer

**Figure 2:**
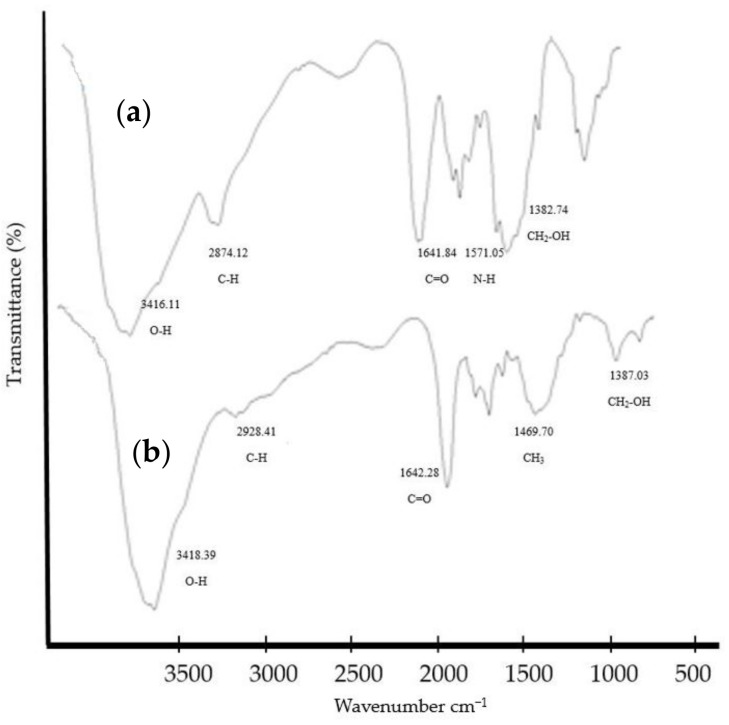
(**a**) The FTIR spectrum of chitosan with its characteristic peaks. (**b**) The FTIR spectrum of synthesized *N*,*N*,*N*-trimethyl chitosan (TMC), approving the *N*-methylation of the polymer.

To confirm the *N*-methylation of the Cs, FTIR spectra were recorded on an FTIR spectrometer in a scan range of 4000–500 cm^−1^. The FTIR spectra illustrated the predominant peaks of Cs and TMC (Figure 2a and 2b, respectively), which are inconsistent with the typical spectra of Cs and TMC reported by previous studies [12,13,14]. The main peaks of Cs are as follows: the absorption peak at 3416 cm^−1^ corresponded to the stretching vibration of O–H and N–H bonds, the absorption peaks at 2874 cm^−1^ due to symmetric stretching vibrations of alkyl (C–H) groups, the absorption peak at 1641 cm^−1^ due to the stretching vibration of carbonyl (C=O) bonds, and the absorption peak of 1571 cm^−1^ assigned to the N–H bending vibration of amino (–NH_2_) groups. Moreover, the absorption peaks around 1155 and 895 cm^−1^ were assigned to C–O–C bending vibration in saccharide repeated units in Cs, and broad peaks at 1091 cm^−1^ corresponded to the C–OH stretching vibration of alcohol on the backbone (Figure 2a). On the other hand, in the FTIR spectrum of TMC, the broad peak corresponded to stretching vibration of both O–H and N–H bonds at 3418 cm^−1^ has less intensity than the same peak in Cs, as it is converted to other groups like –NH(CH_3_), –N(CH_3_)_2_, and –N^+^(CH_3_)_3_. This, in addition to the new peak that appeared at 1642 cm^−1^ related to the –C=O stretching vibration of remaining *N*-acetyl groups, showed the *N*-methylation of Cs. Moreover, a new absorption peak that appeared at 1469 cm^−1^, which corresponded to the bending vibration of the methyl groups of the *N*-alkylated (–NH(CH_3_), –N(CH_3_)_2_, and –N^+^(CH_3_)_3_) groups in TMC, proved the successful synthesis of TMC from Cs (Figure 2b).

**Figure 3:**
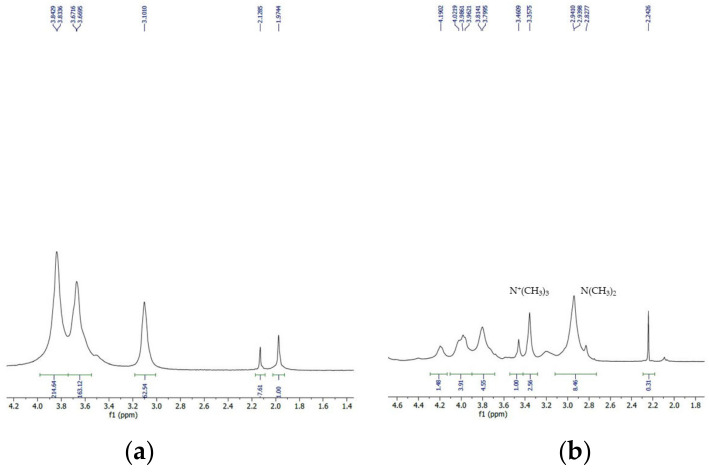
(**a**) ^1^H Nuclear magnetic resonance (^1^H NMR) spectrum of chitosan. (**b**) The ^1^H NMR spectrum of synthesized *N*,*N*,*N*-trimethyl chitosan (TMC), representing a 42.6% *N*-methylation of the polymer.

Additionally, Figure 3a,b represents the ^1^H NMR spectra of Cs and TMC and corresponding peak assignments. The ^1^H NMR spectrum of Cs showed a typical C-1 spectrum at 3.1 ppm, and the spectra of C-2, C-3 to C-6, at 3.6–3.9, relevant to the glucosamine unit skeleton. Additionally, the weak peak at 2.00 was assigned to the residual methyl protons of the *N*-acetyl group of Cs [15]. The introduction of three methyl groups at the primary amino groups of repeating monomers of Cs to form TMC was verified from the ^1^H NMR spectrum of TMC with the appearance of a prominent signal for trimethyl groups at 2.85 and 3.1 relevant to N^+^(CH_2_), and 3.4 ppm relevant to N^+^(CH_3_)_3_ [16]. Besides the FTIR spectra, these data adequately indicated the successful synthesis of TMC from Cs. The degree of quaternization of TMC was calculated to be 42.6%.

### 3.2. Preparation of TMC-NPs

The TMC-NPs were synthesized according to the ionic gelation process using TPP, the cross-linker. The final pH of the TMC-NP solution was recorded as 6.00 ± 0.1. In the optimization steps of TMC-NPs, the higher ratios of TMC/TPP and TMC/insulin resulted in higher zeta potential but larger sizes and a PDI. Similarly, a higher TMC/rivastigmine ratio caused larger sizes but a lower EE. The EE of different formulations for rivastigmine ranged between 35.00% and 90.00%. Among all the TMC-NP formulations, the TMC/TPP weight ratio of 40/1 was selected as the best formulation based on its optimum size, PDI, and zeta potential responses, with the EEs of 58.00 ± 0.5 and 47.8 ± 0.2 for rivastigmine and insulin (mean ± SD), respectively.

### 3.3. Physiochemical Characterization of TMC-NPs

**Figure 4:**
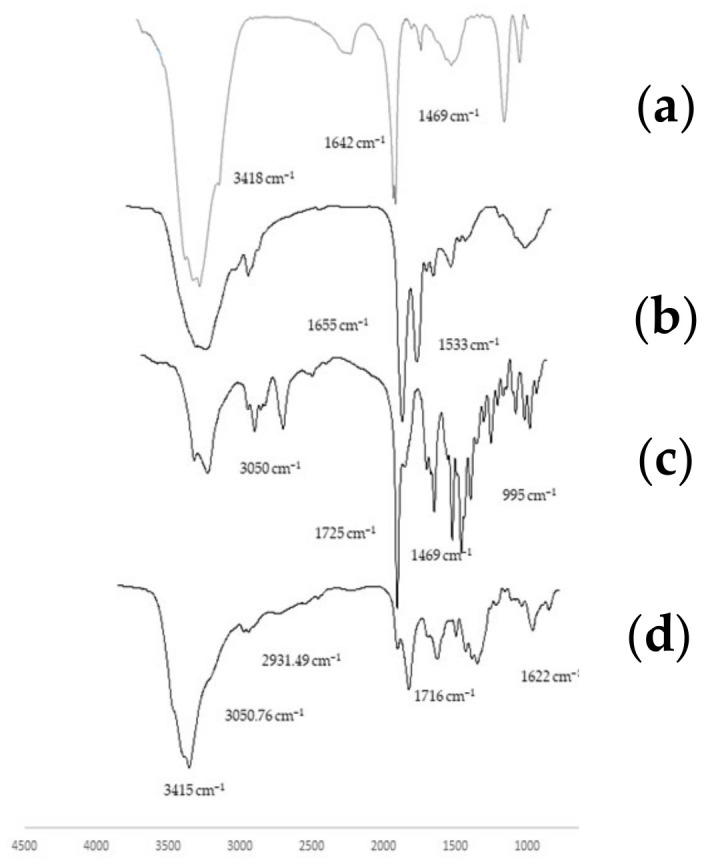
The FT-IR spectra of the following: (**a**) *N*,*N*,*N*-tri methyl chitosan (TMC); (**b**) Insulin; (**c**) rivastigmine; (**d**) Rivastigmine- and insulin-loaded TMC-nanoparticles. The spectrum of the rivastigmine- and insulin-loaded nanoparticles indicates all essential peaks of the pure polymer and both drugs.

Figure 4 displays the FTIR spectra of pure TMC, insulin, and rivastigmine (4a, b, and c, respectively), as well as the rivastigmine- and insulin-loaded TMC-NPs (4d). As mentioned earlier, the FTIR spectrum of TMC showed a weak and broad peak related to the stretching vibration of N–H bonds at 3418 cm^−1^, in addition to the 1642 cm^−1^ peak corresponded to the –C=O stretching vibration of remaining *N*-acetyl groups and a peak at 1469 cm^−1^ related to the bending vibration of methyl groups of *N*-alkylated (–NH(CH_3_), –N(CH_3_)_2_ and –N^+^(CH_3_)_3_). Moreover, the FTIR spectrum of the insulin showed the typical N-H starch spectrum at 3318.69. Two clear shoulder absorptions that appeared at 1655 cm^−1^ and 1533.72 cm^−1^ were related to amide I and amide II, respectively, as depicted in Figure 4b. The 1393.67 cm^−1^ reported peak corresponded to C–O starch bonds in the insulin structure. On the other hand, the chemical structure of the rivastigmine showed absorption bonds in 3408 cm^−1^, which corresponds to N–H stretching. The other predominant peaks of the main functional groups of pure rivastigmine were positioned at 3050.76 cm^−1^, demonstrating the aromatic cycle of the drug structure and the 1725 cm^−1^ peak related to the ester functional group and C=C bands; 1464 cm^−1^ refers to C–N stretching in tertiary amines and 955.79 cm^−1^ corresponds to the =C–H bending. The formation of rivastigmine- and insulin-loaded TMC-NPs changed the FTIR spectrum of both drugs and polymers. As shown in Figure 4d, the recorded peaks at 3415 cm^−1^ and 2931.49 cm^−1^ were related to NH and CH starching bonds, respectively. The 1716.59 cm^−1^ peak is related to the carbonyl groups of TMC. The aforementioned 3050.76 cm^−1^ peak related to the aromatic functional group of rivastigmine with an inconsiderable reduction in peak intensity presented in the FTIR spectra of the NPs due to the incorporation of the drug into the polymer. The specific band at 1622 cm^−1^ can be related to the amid I in insulin. The spectra of NPs also showed all essential peaks of the pure polymer and drugs; however, the broad peaks in TMC spectra, especially in the N-H region, could mask other related peaks in rivastigmine and insulin. The recorded FTIR spectra of the rivastigmine and insulin are similar to the former studies [17,18].

**Figure 5:**
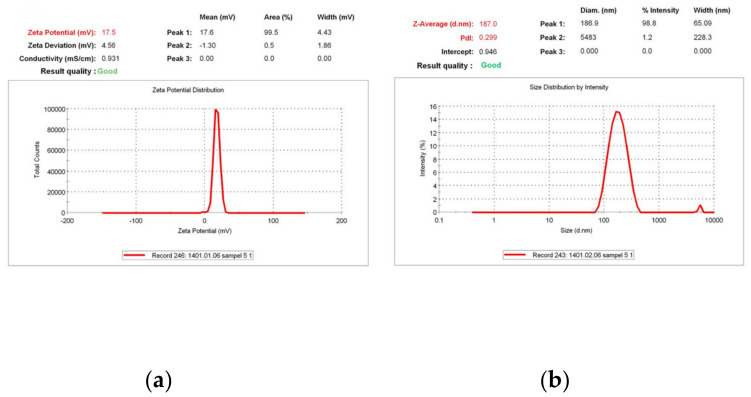
Dynamic Light Scattering (DLS) results showed the following: (**a**) A zeta potential of +17.6 mV for *N*,*N*,*N*- trimethyl chitosan NPs entrapping both rivastigmine and insulin; (**b**) A dynamic diameter of 187.00 nm and polydispersity index of 0.299.

**Figure 6:**
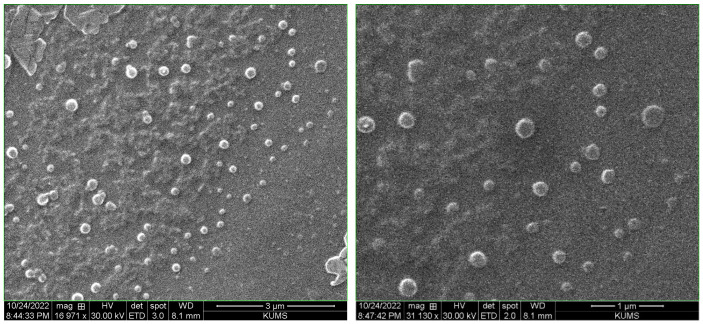
Scanning electron microscopic (SEM) images of rivastigmine- and insulin-loaded *N*,*N*,*N*-trimethyl-chitosan nanoparticles validating the uniform, spherical shape of the nanoparticles with narrow size distribution.

**Figure 7:**
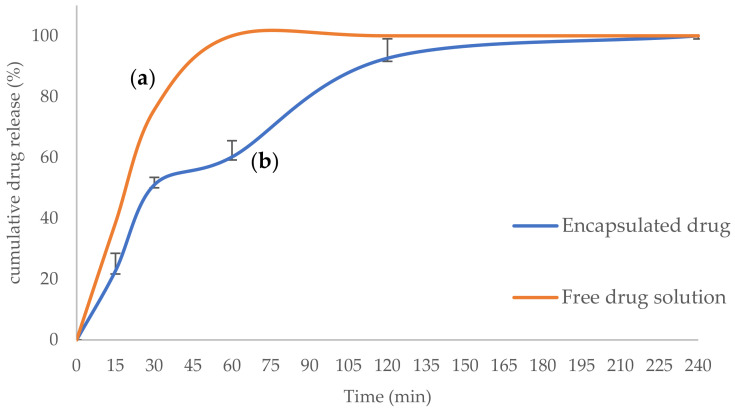
Cumulative in vitro drug release for the following: (**a**) Free rivastigmine (orange); (**b**) Encapsulated rivastigmine in the *N*,*N*,*N*-trimethyl chitosan nanoparticles (blue).

The DLS results showed the zeta potential of +17.5 mV for synthesized NPs (Figure 5a), granting their mucoadhesive properties due to the affinity to negative-charged mucin in the nasal cavity. Additionally, the mean particle size and PDI of the NPs were 187.00 nm and 0.299, respectively (Figure 5b), which is a suitable size for nasal penetration and good quality of the PDI for the stability of the NPs against aggregation. As expected, the particle size and PDI of rivastigmine- and insulin-loaded TMC-NPs were verified using the SEM (Figure 6). Next, the synthesized TMC-NPs were evaluated for in vitro release of rivastigmine. The in vitro release profile of the rivastigmine solution and the optimized TMC-NPs is shown in Figure 7. The in vitro cumulative release from TMC-NPs was diagrammed against time for 4 h and compared with the free drug release from a dialysis bag (14 kD) in the same time intervals. Our findings showed more than 60% of the rivastigmine solution was released from the dialysis bag over the first 15 min. On the other hand, the release of rivastigmine from the TMC-NPs was a gradual release over 4 h up to 99% within this time interval, representing a biphasic drug release pattern.

### 3.4. Ex Vivo Diffusion of Drug Solutions and TMC-NPs

**Figure 8:**
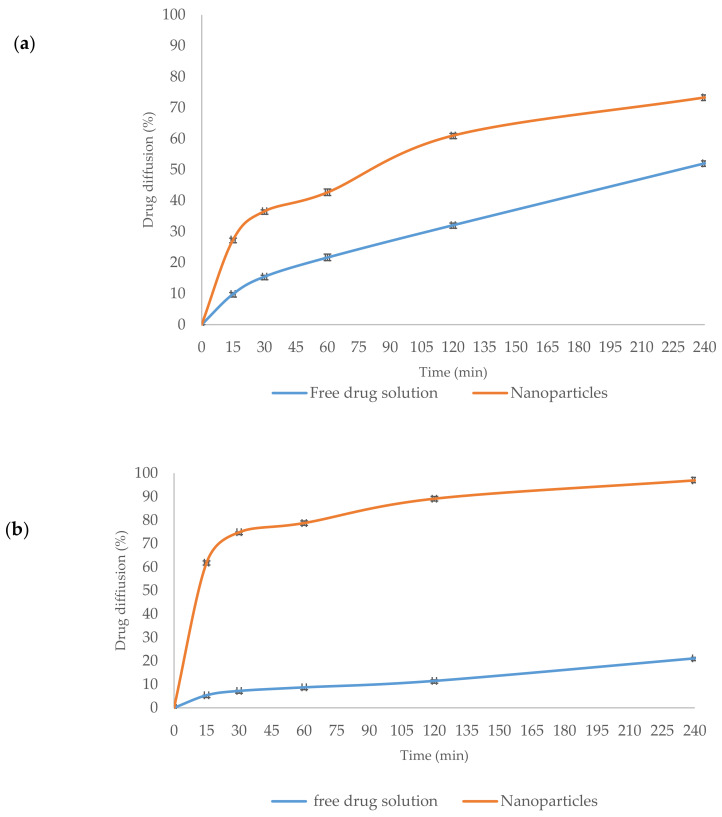
The diffusion profiles of the following: (**a**) Free rivastigmine (blue) in comparison to loaded rivastigmine into *N*,*N*,*N*-trimethyl chitosan nanoparticles (orange); (**b**) Free insulin (blue) in comparison to loaded insulin into *N*,*N*,*N*-trimethyl chitosan nanoparticles.

Ex vivo diffusion of rivastigmine and insulin from free drug solutions and TMC-NPs is shown in Figure 8a, b. It was observed that the diffusion of insulin and rivastigmine drug solutions across the nasal mucosa was small. Almost 52% and 21% in the case of rivastigmine and insulin drug solutions diffused in 4 h, respectively, while in the case of rivastigmine- and insulin-loaded TMC-NPs, higher diffusion was observed for rivastigmine (73%) and insulin (96%). Noticeably, the difference between the solution of the drug and NPs in the case of insulin is significantly higher than rivastigmine, which indicates the greater role of the NPs in improving the penetration of the insulin than rivastigmine. The results for rivastigmine and insulin diffusion over time across nasal mucosa showed a significant increase in diffusion efficacy for both drugs when encapsulated in TMC-NPs.

### 3.5. Biocompatibility Evaluation of TMC-NPs

**Figure 9:**
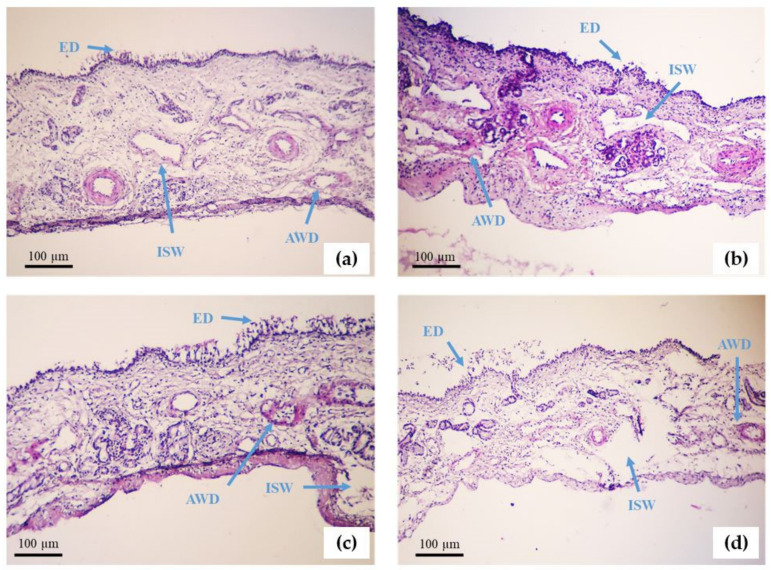
Sheep nasal epithelium tissue after 1 h incubation in the following: (**a**) Negative control (NC); (**b**) Positive control (PC); (**c**) Rivastigmine- and insulin-loaded *N*,*N*,*N*-trimethyl chitosan nanoparticle; (**d**) The mixture solution of free rivastigmine and insulin, all stained with hematoxylin-eosin and observed under a light microscope (×10 magnification). Blue arrows show epithelial detachment (ED), arterial wall damage (AWD), venule wall damage (VWD), and intercellular space widening (ISW).

Figure 9 shows the optical microscope images of histological sections of sheep nasal epithelium samples after treatment with the NC, PC, solutions of drugs, and NPs stained with HE. The sheep nasal epithelium tissue after 1 h incubation in each sample showed a distinct pattern of tissue damage. The highest tissue damage is indicated in the nasal epithelium incubated in the PC group, followed by the drug solution group. However, in the case of nasal mucosa treated with the TMC-NP group, the tissue was preserved in comparison to all other groups, assuring the safety and biocompatibility of TMC-NPs for the nasal epithelium. Table 1 shows the biocompatibility statistics of the NP samples in comparison to other groups. In all four groups, the tissue damage was recorded after the study. However, the post hoc results indicated for ED, the differences between all the groups were negligible except for the PC and the NPs (*p* = 0.035). Additionally, for arterial wall damage (AWD), the PC group is significantly different from the other three groups, and for venule wall damage (VWD), the assessment demonstrated that the drug solution group was similar to the PC to some extent, but the NP group was similar to the NC. Moreover, the ISW assessment indicated the similarity between the NC and NP groups and the difference between these two with drug solution and PC groups. Thus, these results implied that the TMC-NPs were safe for nasal administration.

## 4. Discussion

In this study, TMC-NPs were synthesized using the ion gelation method and characterized to develop an optimized formulation for intranasal drug delivery. While Cs exhibits excellent characteristics as a biopolymer in the pharmaceutical field, its limited solubility in aqueous media restricts its applicability [48]. However, derivatization of Cs to TMC retains the inherent outstanding properties of Cs while improving its solubility in aqueous, acidic, and alkaline solutions [15,49]. In this study, we successfully synthesized TMC for intranasal purposes, which is consistent with previous studies [50,51,52]. Furthermore, we observed that the formation of rivastigmine- and insulin-loaded TMC-NPs resulted from the ionic interaction between the positively charged amino groups of TMC and the negative groups of TPP and insulin, which entrap small molecules of rivastigmine. The higher TMC/TPP weight ratio used in the synthesis of TMC-NPs, compared to other studies using the ion gelation method [7,53,54], can be attributed to the presence of insulin with a negative surface charge, which promotes polyelectric interactions between the TMC polymer and insulin instead of TPP [2]. Moreover, the rivastigmine- and insulin-loaded TMC-NPs were synthesized with a final solution pH of 6.00 ± 0.1, which is suitable for intranasal administration, considering that the natural pH of the nasal cavity ranges from 5.5 to 6.5 [55].

This study demonstrated the successful encapsulation of both rivastigmine and insulin within TMC-NPs, achieving the desired size, polydispersity index (PDI), and zeta potential for effective intranasal drug delivery. In the context of intranasal administration, the size and zeta potential of NPs play crucial roles in the success of drug delivery systems [56]. The size and PDI of NPs influence their ability to penetrate the mucus layer, interact with the nasal epithelium, and facilitate efficient cellular internalization [57]. Smaller NPs (up to 200 nm) offer advantages such as improved mucus penetration and prolonged residence time in the nasal epithelium [58]. On the other hand, maintaining an appropriate zeta potential of NPs enhances their stability, mucoadhesive properties, and cellular internalization within the nasal mucosa [59]. Striking a balance between NP size and zeta potential is vital to achieving optimal drug delivery outcomes, ensuring efficient absorption and maximizing therapeutic efficacy in the intranasal route [60].

The ex vivo experiment revealed that the release behavior of the encapsulated drugs did not exhibit a biphasic pattern observed in the in vitro release study. This observation can be attributed to the characteristics of the nasal mucosa, which act as rate-limiting barriers for drug diffusion from the NPs. Over time, the diffusion of rivastigmine and insulin across the nasal mucosa demonstrated a significant increase in efficacy when they were [encapsulated in TMC-NPs. This difference is likely attributed to the well-known diffusion-enhancing properties of TMC in overcoming mucosal obstacles [61]. Quantitative analysis indicated that the amount of rivastigmine and insulin permeating through the nasal mucosa and collected in the acceptor buffer solution at the end of the experiment was approximately 1.4-fold and 4.6-fold higher, respectively, compared to drug solutions. These findings from the ex vivo permeation experiment suggest that TMC-NPs can enhance the diffusion of both drugs across the sheep nasal mucosa, allowing them to reach their targeted therapeutic doses in the brain. Furthermore, histological studies demonstrated that TMC-NPs can improve the delivery of both drugs without causing significant toxicity to the nasal tissue. Therefore, TMC-NPs hold an interesting potential as an intranasal drug delivery system, providing longer residence time and enhanced diffusion across the nasal membrane.

## 5. Conclusions

In summary, our findings demonstrate significantly improved drug transport efficiency through sheep nasal mucosa using the NPs compared to drug solutions. The NPs exhibited transport efficiencies for rivastigmine and insulin that surpass the efficiencies of the individual drug solutions. These results highlight the potential of a new drug delivery system as a promising approach for enhancing nasal transport efficiency and that combinational mucoadhesive NPs may offer a novel strategy for the simultaneous cerebral delivery of rivastigmine and insulin. This could prove helpful for developing new effective treatments of Alzheimer’s disease and other neurodegenerative conditions.

## Figures and Tables

**Table 1:** The lesion frequencies of the sheep nasal epithelium tissues incubated in the rivastigmine and insulin-loaded *N*,*N*,*N*-trimethyl chitosan nanoparticle sample, negative control, and positive control.

Tissue Damage	PC	NC	DS	TMC-NPs
Epithelial detachment	2.9259 ± 0.19 ^a^	2.5106 ± 0.11 ^a^	2.3500 ± 0.24 ^a^	2.3000 ± 0.14 ^a^
Arterial wall damage	2.8889 ± 0.15 ^b^	1.6383 ± 0.10 ^a^	1.8947 ± 0.21 ^a^	1.6400 ± 0.8 ^a^
Venules wall damage	3.8148 ± 0.076 ^b^	1.2553 ± 0.06 ^a^	2.7000 ± 0.17 ^ab^	1.9200 ± 0.14 ^a^
Intercellular space widening	3.1852 ± 0.16 ^b^	2.5106 ± 0.12 ^a^	3.3500 ± 0.23 ^b^	2.3600 ± 0.13 ^a^

PC: positive control, NC: negative control, DS: Drug solution, TMC-NPs: rivastigmine- and insulin-loaded *N*,*N*,*N*-trimethyl chitosan nanoparticles. Rows with different superscripts show significant differences among groups.

## Data Availability

Data is contained within the article.
